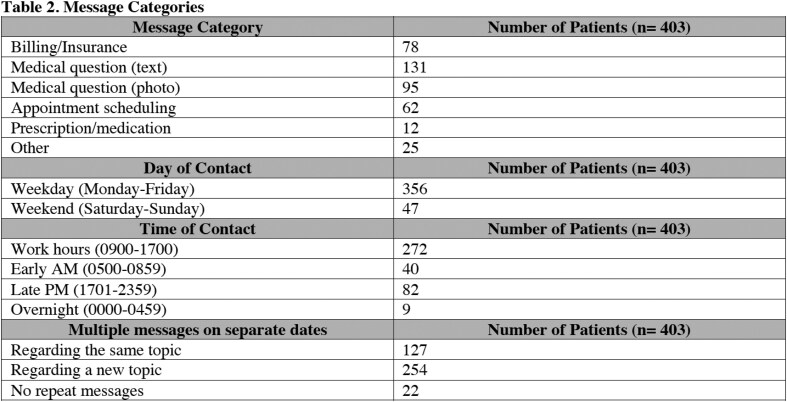# “Doc, Is This Normal?” A Retrospective Review of Patient Portal Messaging for a Single Surgeon in an Academic Plastic Surgery Practice

**DOI:** 10.1093/asjof/ojag138.001

**Published:** 2026-07-24

**Authors:** Jessica Berns, Justin L Anderson, Detlev Erdmann

**Affiliations:** Division of Plastic, Maxillofacial, and Oral Surgery, Department of Surgery, Duke University Medical Center, Durham, NC, USA; Duke University School of Medicine, Durham, NC USA; Division of Plastic, Maxillofacial, and Oral Surgery, Department of Surgery, Duke University Medical Center, Durham, NC, USA

## Abstract

**Goals/Purpose:**

Secure messaging increases patient engagement but adds to physician workload and burnout. Plastic surgeons face heightened pressure to maintain responsiveness and satisfaction due to online ratings. This study examines EMR messaging trends to optimize discharge and preoperative information to reduce message volume and improve patient-provider communication while supporting physician well-being in a highly scrutinized field. Methods/Technique A single-institution, IRB-approved retrospective review was conducted of patient messages directed to a single academic plastic surgeon at Duke University Hospital over a span of 18 months from July 1, 2023, through December 31, 2024. Patient demographics, message topics, and the date/time of each message were extracted via chart review then securely stored in a REDCap database for subsequent analysis.

**Methods/Technique:**

A single-institution, IRB-approved retrospective review was conducted of patient messages directed to a single academic plastic surgeon at Duke University Hospital over a span of 18 months from July 1, 2023, through December 31, 2024. Patient demographics, message topics, and the date/time of each message were extracted via chart review then securely stored in a REDCap database for subsequent analysis.

**Results/Complications:**

Of the 403 patient-initiated EMR messages, most were from women (343) aged 30–50 (171). Over half (213) were preoperative and related to aesthetic body procedures (154). Medical questions with and without photos were the most frequently asked questions. Message frequency peaked on weekdays during work hours.

**Conclusion:**

This review of patient-initiated messages to an academic plastic surgeon over a continuous 18- month period indicates that digital communication has become a significant component of clinical workflow with most inquiries from middle-aged female patients seeking aesthetic procedures, often preoperatively and during already busy work hours. Optimizing patient education, triage protocols, and institutional support (via office staff, RN;s, and PA’s/NP’s) in the preoperative period can reduce unnecessary queries, improving communication efficiency while balancing patient needs with physician well-being.

**Figure:**
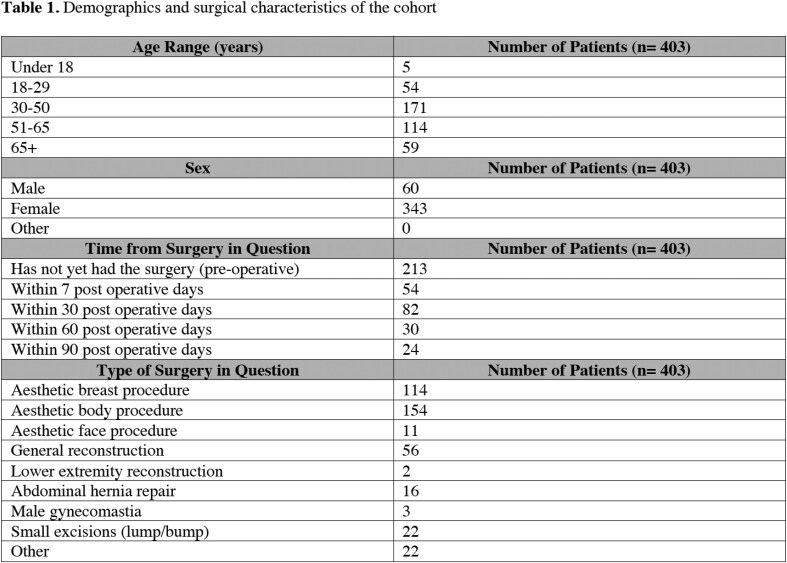

**Figure:**